# Study of *Leishmania* pathogenesis in mice: experimental considerations

**DOI:** 10.1186/s13071-016-1413-9

**Published:** 2016-03-11

**Authors:** Corinne Loeuillet, Anne-Laure Bañuls, Mallorie Hide

**Affiliations:** Maladies Infectieuses et Vecteurs: Ecologie, Génétique, Evolution et Contrôle (MIVEGEC), UMR IRD 224-CNRS 5290, Institut de Recherche pour le Développement (IRD), Centre National de la Recherche Scientifique (CNRS), Université de Montpellier, Montpellier, France

**Keywords:** Leishmaniases, Visceral, Cutaneous, Mice, Dose, Inoculation site, Saliva

## Abstract

Although leishmaniases are endemic in 98 countries, they are still considered neglected tropical diseases. Leishmaniases are characterized by the emergence of new virulent and asymptomatic strains of *Leishmania* spp. and, as a consequence, by a very diverse clinical spectrum. To fight more efficiently these parasites, the mechanisms of host defense and of parasite virulence need to be thoroughly investigated. To this aim, animal models are widely used. However, the results obtained with these models are influenced by several experimental parameters, such as the mouse genetic background, parasite genotype, inoculation route/infection site, parasite dose and phlebotome saliva. In this review, we propose an update on their influence in the two main clinical forms of the disease: cutaneous and visceral leishmaniases.

## Background

Leishmaniases are caused by pathogens of the genus *Leishmania* and are characterized by an important clinical and epidemiological diversity. According to the World Health Organization (WHO), leishmaniases occur in four continents and are endemic in 98 countries [1]. The causative parasites are classified according to genetic, biological and immunological criteria. More than 30 *Leishmania* spp. are known of which 20 are pathogenic for humans (for a taxonomic table, see [2]). In humans, this disease can have several clinical manifestations of variable severity: mucosal leishmaniasis (a mutilating disease), diffuse cutaneous leishmaniasis (a long-lasting disease due to a deficient cell-mediated immune response), cutaneous leishmaniasis (disabling with multiple lesions) or visceral leishmaniasis, which is fatal if untreated (see for reviews [3, 4]). Leishmaniasis can also be asymptomatic in humans [5, 6] and in animal reservoirs [7, 8]. It is worth noting that a single species of *Leishmania* may cause several disease forms [3].

Drug-resistant *Leishmania* isolates or emerging virulent strains are often described [9, 10]. It is crucial to understand how these new strains disseminate and are controlled by the host immune system to fight against them. To investigate the pathogenic diversity, the impact of the host genetic background and of the *Leishmania* genotypes, animal models are widely used. Classically, in infected animals, parasite-activated CD4^+^ T cells rapidly proliferate in the lymph nodes, differentiate and secrete specific cytokines. Th1 cells secrete IL2, IFNγ and TNFα, leading to macrophage activation and parasite elimination. On the other hand, the Th2 response is associated with IL4, IL5 and IL13 production and with parasite proliferation (for review see [11]). When studying a newly isolated strain, the experimental settings have to be carefully designed and several parameters must be taken into account. The objective of this review is to summarize results on the pathogenic mechanisms in mice infected by *Leishmania* spp. We will focus on the two main clinical forms: visceral leishmaniasis (VL) and cutaneous leishmaniasis (CL). We will first describe the experimental data on the influence of the genetic background in mouse models of VL and CL caused by *Leishmania donovani* and *L. infantum* and of CL caused by *L. major*, *L. mexicana* and *L. tropica*. Then, data obtained in mouse models of VL by *L. infantum* and of CL by *L. major* will be reviewed, particularly: (i) the immune cells involved and the associated-immune response and (ii) the parameters (mouse and parasite genotypes, parasite dose and inoculation route) that influence the infection outcome.

## Review

### Visceral leishmaniasis

Human VL is the most severe form of this disease and occurs when infected people are left untreated. Ninety percent of reported cases of VL are found in Bangladesh, Brazil, India, Nepal and Sudan (WHO). Approximately, 0.2 to 0.4 million cases of VL are reported each year and VL causes 20,000 to 40,000 deaths annually [1]. It is caused by parasites of the *Leishmania donovani* complex: *L. donovani*, *L. archibaldi* and *L. infantum* (syn. *L. chagasi*). The main causative agents of VL in humans are *L. donovani* and *L. infantum*, whereas *L. infantum* can cause CL (see below) and is the main VL-causing parasite in dogs, which are considered to be *Leishmania* spp. reservoirs [3]. Hereafter, we will describe the main experimental parameters that need to be taken into account when studying visceralizing *Leishmania* strains.

### Mouse genetic background

Susceptibility and resistance to *Leishmania* infection in humans and in mice are regulated by genetic determinants [12]. In the mouse, two main loci have been described: *Lsh* and *H*2 (the major histocompatibility complex). The *Lsh* locus on mouse chromosome 1, with *Nramp1* (*Slc11a1*) as the main candidate gene [13], influences the natural resistance to *L. donovani* infection (control of parasite load in liver and spleen) [14]. In mice harboring wild type *Scl11a1*, such as the CBA mouse strain, parasite proliferation in liver is hindered. Conversely, in mice with mutant *Scl11a1*, such as the BALB/c and C57BL/6 strains, parasite growth is unrestrained [15]. The *H*2 locus [16] is involved in the development of the adaptive immunity and overcomes the innate susceptibility to *L. donovani* caused by *Scl11a1* mutations [15]. Like for *L. donovani*, *L. infantum* primary infection is also initially controlled by the *Lsh* locus and then by the *H*2 locus. Both loci are involved in the development of the acquired immune response [17]. For instance, *L. infantum* susceptible mice (with mutant *Scl11a1*) harboring the H2-b or H2-r alleles, show a “cure” phenotype associated with a strong reduction of the parasitic load in liver. Conversely, H2-d, H2-q and H2-f susceptible mice are characterized by a “non-cure” phenotype. Mice can thus be classified in four phenotypic categories: resistant, susceptible and when susceptible, in cure and non-cure (Table 1). Non-cure mice, such as the BALB/c strain, will spontaneously progress to a chronic phase of the disease without total parasite clearing. Conversely, in cure mice, such as the NMR1 strain, the parasite load is very low and parasites can be fully eliminated in some cases.

**Table 1:** Influence of the mouse genetic background on visceral leishmaniasis outcome

Locus	Phenotype	Mouse strain	Reference
Lsh	resistant	NMR1, A2G, HR, Ash, CD2, CBA	[14, 15, 17]
Lsh	susceptible	B10D2, C3H, PO, BALB/c, C57BL/6	[14, 15, 17]
Lshs/H2^k/b^; H2^r^	cure	B10.A4R	[17]
Lshs/H2^d^; H2^q^; H2^f^	non-cure	B10.HTG, B10.D2/n	[12, 17]

In conclusion, it is clear that the mouse genetic background influences the *Leishmania* infection outcome and this feature must be taken into account when designing experiments. Although BALB/c mice will not die of VL (differently from untreated humans), they can be used to study the immunopathology changes occurring during VL. Indeed, they present clinical features of human VL, such as hepatosplenomegaly or disruption of splenic tissue. However, the infection outcome depends also on the ability of the infected mice to induce a good Th1 immune response as well as on the inoculation route and injected dose, as discussed hereafter.

### Immune control of infection

From an immunological point of view, the main feature of VL is its organ specificity. Indeed, studies on VL progression in mice highlighted that the major responding tissues (spleen and liver) present distinct patterns in term of immune response and parasite control. When injected intravenously in BALB/c mice, more than 95 % of *L. infantum* promastigotes are rapidly cleared from the circulation by highly phagocytic macrophages of the spleen marginal zone [18]. After pathogen uptake, macrophages exert their leishmanicidal activity through nitric oxide (NO) synthesis. During the course of infection, the parasite burden is progressively controlled [19] with a peak of parasitemia at week 8 of infection [20]. In the spleen, the course of parasite burden reflects the cytokine production kinetics and the activation of the different classes of immune cells. At the beginning of infection (first 4 weeks), parasite replication is associated with the immune cell inability to produce IFNγ and IL2 (macrophage-activating cytokines), whereas production of IL4 or IL5 is conserved [21, 22]. Conversely, IL10 and TGFβ (macrophage-inhibitory cytokines) are produced by cells of the spleen marginal zone and of the red pulp, respectively. IL10 and TGFβ are thought to contribute to the establishment of infection and parasite replication [19]. After the first 4 weeks of infection, CD4^+^ T, CD8^+^ T and natural killer (NK) cells recover their capacity to produce IFNγ, thus promoting the macrophage microbicidal activity with NO synthesis and control of granuloma formation in liver (see next paragraph) and ultimately parasite burden reduction [21]. In synergy with IFNγ, IL17A also contributes to macrophage activation with NO production, leading to parasite clearance [23]. Nevertheless, infection in the spleen is maintained during the entire VL course. This parasite persistence may be due to sustained TGFβ production by CD4^+^ CD25^+^ T cells (Treg) that contributes to immunosuppression [24].

In liver, the infection time course is different. After injection in the lateral tail vein, promastigotes invade the resident macrophages, Kupffer cells and dendritic cells where they become amastigotes and replicate. During acute infection (first 2 weeks), parasite growth is uncontrolled, due to elevated TGFβ levels and ineffective Th1 response [25]. This correlates with the peak of parasite burden observed between 2 and 8 weeks post-infection. Liver infection is then resolved through the formation of granulomas that are characterized by parasitized Kupffer cells surrounded by a mantle of lymphocytes [26]. Finally, after 8 weeks of infection, amastigotes are almost absent in granulomas and infection is resolved [26].

In VL, the organ-specific immunity with elimination of parasites in liver and their persistence in the spleen directly reflects the observed immune response. This chronic status is critical because the host becomes more susceptible to secondary infections. Thus, to develop and evaluate new vaccines or therapies against leishmaniases, it is crucial to determine the role of each immune cell type in the establishment of the cellular immune interplay resulting in the control of the infection.

For VL, a thorough understanding of the mechanisms underlying the protective immune response in liver and the failure in spleen would allow the development of new candidate vaccines and of new strategies or treatments to eliminate the parasite in infected people.

### Tissue of origin and parasite genotype

Another question is whether the disease pattern differs depending on the tissue of origin of the parasites used to infect mice (isolated from cutaneous or visceral forms in humans). Comparison of the infection profiles (parasite burden in spleen and liver) in BALB/c mice of 22 *L. infantum* strains isolated from patients with CL or VL revealed a great variability of infection profiles (progressive, controlled or undetectable infection) [27]. The parasite zymodeme and the host immune status did not have any influence on the infection profile. However, no strain of cutaneous origin caused a visceralizing form in mice [27]. In susceptible mice, histopathological analysis of the liver revealed no difference whatever the *L. infantum* strain used, although a more pronounced liver granulomatous response was observed with visceralizing strains [28]. Interestingly, the visceralizing and infection profiles were preserved in BALB/c and C57BL/6 mice, both susceptible to infection with a non-cure and a cure profile respectively, suggesting a clear role of parasite factors on the infection outcome. This was confirmed by the finding that the infection kinetics were similar following experimental infection of immunodeficient C.B-17 SCID and congenic BALB/c mice, although the parasite load was higher in immunodeficient animals [29].

Moreover, *L. infantum* strains belonging to the same zymodeme (MON-1) can lead to different infection profiles in mice. The most pathogenic strains induced splenomegalia and higher parasite load in spleen and liver associated with higher IgG1, TGFβ and reduced IFNγ production [30]. This effect was not related to the host species (dog or human) from which the parasites were isolated [30]. These observations highlight the intra-strain specific virulence variability and confirm that in VL, parasite virulence is a clonal or inbred dominant trait within each strain (infection study of 11 clones derived from three strains of known virulence) [31].

The influence of the host immune status on the strain virulence is not well known. Indeed, strains isolated from immunosuppressed patients display either high [27] or low virulence [30].

In conclusion, it is difficult to extend experimental results in mouse models obtained with a given *Leishmania* strain to other strains even if they are genetically related or responsible for the same clinical form. Therefore, it is crucial to analyze all new parasite isolates and the corresponding immune response in mice. Such studies are particularly recommended for parasite stocks that cause large epidemics or non-pathogenic and silent leishmaniasis forms in humans. Prospective studies in endemic areas also are important to better understand the basis of the protective immune response in humans and to develop specific and more efficient treatments.

### Inoculation route and parasite dose

Several inoculation routes have been tested (Table 2). It appears that the parasite load (reflecting the immune response strength) depends not only on the parasite dose that is inoculated, but also on the chosen injection route.

**Table 2:** Inoculation route and parasite dose influences on visceral leishmaniasis outcome in susceptible BALB/c mice

Infection route	Parasite dose	Parasite strain	Liver load	Spleen load	Th1 response	Th2 response	Reference
Subcutaneous	10^5^	LIVT-1	+	-	ND	ND	[32]
	10^6^		+	+	ND	ND	
	10^7^		+	+	ND	ND	
	10^3^	MHOM/BR/1970/BH46	+/-	++	+	+	[33]
	10^5^		+	+++	+	+	
	10^7^		++	++++	+	+++	
	10^3^	MHOM/IN/80/DD8	+/-	ND	+++	+	[34]
	10^5^		+	ND	++	+	
	10^7^		+	ND	++	++	
Intradermal	10^3^	MHOM/IN/80/DD8	+	ND	+++	+	[34]
	10^5^		++	ND	++	+	
	10^7^		++	ND	++	+	
	10^7^	MHOM/ES/92/LLM-320	+++	+++	+	+	[38]
Intraperitoneal	10^3^	MHOM/IN/80/DD8	++	ND	++	++	[34]
	10^5^		+++	ND	+	++	
	10^7^		+++	ND	+	+++	
	10^7^	MCAN/PT/94/IMT205	ND	+	ND	ND	[37]
Intracardiac	10^3^	MHOM/IN/80/DD8	++	ND	++	++	[34]
	10^5^		+++	ND	+	++	
	10^7^		++++	ND	-	+++	
	10^7^	MHOM/IN/1993/B12302	+++	++	++	++	[35]
Intravenous	10^5^	LIVT-1	+	+	ND	ND	[32]
	10^6^		++	+	ND	ND	
	10^7^		+++	+	ND	ND	
	10^7^	MHOM/BR/1970/BH46	+	+	+	++	[33]
	10^3^	MCAN/ES/96/BCN150	+	+	+/-	-	[36]
	10^5^		++	++	+/-	+/-	
	10^6^		++	+++	+	++	
	10^4^	MHOM/ES/92/LLM-320	+/-	+	ND	ND	[38]
	10^5^		+	++	ND	ND	
	10^6^		++	+++	ND	ND	
	10^7^	MCAN/PT/94/IMT205	ND	+	ND	ND	[37]

*ND* not done

For the LIVT-1 strain, the subcutaneous route seems to be less efficient (based on the parasite load in the spleen) than the intravenous one, when mice are inoculated with medium doses of parasites (10^5^); however, it has been not demonstrated for higher doses (10^6^ and 10^7^) [32]. Moreover, the parasite load in the liver is higher following intravenous inoculation compared to the subcutaneous route and the response is dose-dependent [32].

A clear dose effect on parasite load was observed in BALB/c mice inoculated subcutaneously with *L. infantum* [33]. Mice inoculated with low doses (10^3^) showed a minimal infection associated with a Th1 response (IFNγ and NO production in spleen), whereas high doses (10^7^) led to high parasite burden in spleen and lymph nodes as well as to a Th2 response [33].

By assessing several inoculation routes, Kaur et al. showed that the subcutaneous route is less efficient than the intradermal, intraperitoneal and intracardiac routes in term of liver parasite load [34]. Again, a clear dose effect on parasitemia was detected. High doses inoculated by intracardiac injection led to the highest liver parasite load and were associated with a Th2 immune response [34]. However, the Th1 immune response necessary for the establishment of resistance in BALB/c mice was strongest when mice were inoculated with low doses of parasites via the subcutaneous or intradermal routes. This was associated with maximum IFNγ production and high level of IgG2a [33, 34]. It is worth noting some discrepancies in studies assessing the same dose and the same route. For example, differently from Rosypal et al., Oliviera and colleagues demonstrated that, at high dose (10^7^), parasite load is higher when mice are inoculated subcutaneously rather than intravenously. Overall, it is difficult to compare different studies because of the different parasite strains and parasite load determination methods. This stresses again that results obtained with a given stock cannot be fully transposed to another one. Moreover, although all the studies summarized here were performed using BALB/c mice, we cannot assume that the animals were genetically identical and this could account for the different responses sometimes observed after *Leishmania* infection.

The intracardiac route is responsible for the development of Th2 immune response that is characterized by IL4 production and increased levels of IgG1 and is associated with IL10 production by Treg cells, thus allowing the establishment of a persistent infection [24, 34]. Although another study described a quite different immune response after intracardiac infection [mixed activating (IFNγ and TNFα)/deactivating (TGFβ) cytokine response] [35], this route seems to be effective in term of infection persistence. Indeed, intracardiac inoculation leads to progressive VL with parasite persistence in the spleen 4 months after the infection and accompanied by massive splenomegaly [35].

Intravenous inoculation seems to lead to effective infection (based on parasite load) whatever the dose used, with parasite persistence in spleen and liver when high doses are injected [36]. Liver lesions are prominent in intravenously inoculated mice, whereas they are almost inexistent in mice injected subcutaneously. However, heterogeneity is observed among animals as already described for spleen parasite load [37]. Liver granuloma formation seems to depend on the dose used: high numbers of mature and sterile granulomas are observed when mice are inoculated with low doses, whereas no parasite clearance is detected after injection of high doses [36]. However, it is worth noting that only mice inoculated with low doses could mount a protective response in the liver and bone marrow, associated with parasite clearance.

Intradermal inoculation of high parasite doses (10^7^) leads to chronic infection with parasite persistence in spleen and lymph nodes, Th1/Th2 cytokines production (IFNγ, IL4) and partial clearance in the liver [38]. When inoculated with low (10^3^) or medium (10^5^) doses, mice are effectively infected, but present a lower parasite load and a Th1-dominant immune response [33].

In conclusion, although the subcutaneous or intradermal routes mimic the natural infection routes, they require high doses of parasites to cause a good and persistent infection. Lower doses can be recommended for vaccination protocols because the generated immune response seems to be sufficient for long-term protection. One study reported that the intraperitoneal route leads to higher homogeneity of infection in term of parasite load and thus might be more appropriate to study new isolates [37]. Conversely, the intravenous route leads to heterogeneous parasite loads among animals and, consequently, errors in the interpretation of the results could occur when analyses are performed on pooled animals.

### Influence of phlebotome saliva

*Leishmania* parasites are classically injected in the skin together with saliva by an infected sandfly during a blood meal. For VL, studies on phlebotome saliva are mainly focused on its modulatory effect on the immune response or as a vaccine candidate rather than on its influence on the primary infection outcome (for review see [39]). To our knowledge, only two studies reported that, differently from CL (see below), salivary extracts do not to have a significant role in VL establishment in dogs and mice [38, 40]. Dogs intradermally inoculated with *L. chagasi* stationary promastigotes together with or without *Lutzomyia longipalpis* salivary gland extract did not show any infection, suggesting that the presence of salivary proteins is not sufficient for effective infection through the intradermal route [40]. In BALB/c mice, intradermal injection of *L. infantum* promastigotes with or without sandfly salivary extracts led to effective visceralization in both cases, showing that salivary products do not influence the infection course (parasite burden in spleen, liver and lymph nodes) [38]. Further studies are needed to validate these results and to determine the potential effect of sandfly salivary components on *L. infantum* visceralization capacity and infection outcome.

In conclusion, when studying VL in mice, it is important to consider the parasite dose as well as the route of inoculation because they clearly influence the development of the T helper response and consequently the infection outcome. The study of the chronic phase of infection requires an inoculation route (intravenous or intradermal) that allows the long-term establishment of the infection. Conversely, for studying the acute phase of infection, the inoculated dose needs to be precisely controlled because a more intense immune response is observed with high doses. For vaccination studies, low doses of parasites (10^4^) can be used because the elicited immune response can protect on the long term.

### Cutaneous leishmaniasis

The incidence of human cutaneous leishmaniasis (CL) is of about 0.7 to 1.2 million cases each year [1] (WHO, 2014). According to the WHO, 90 % of CL cases occur in Afghanistan, Brazil, Iran, Peru, Saudi Arabia and Syria. It is usually a self-healing disease, but in the presence of multiple lesions, CL can lead to lifelong aesthetic stigma [4]. CL is caused by several *Leishmania* species: *L. major*, *L. tropica*, *L. mexicana*, *L. amazonensis*, *L. braziliensis* and *L. guyanensis* [2, 3]. *L. infantum*, a classically visceralizing species, can also present an unusual skin tropism, thus rarely causing limited cutaneous lesions at the biting site [41].

### Mice genetic background

Studies on the infection phenotype in mice with different genetic background and in inbred congenic mice have allowed the identification of several loci involved in the infection control (Table 3). Analysis of the lesion size in the progeny from crosses between resistant and susceptible mice following intradermal infection with *L. tropica* led to the hypothesis that susceptibility (C57BL/6 x Balb/c) or resistance (C3H/HeN x P/J) to CL could be controlled by a unique locus with minor influences by other genes [42, 43]. However, the finding that not all animals with cutaneous lesions will develop a systemic infection suggests that different genes may be involved in this two forms of disease [42, 43].

**Table 3:** Influence of the mouse genetic background on cutaneous leishmaniasis outcome

Locus	Phenotype	Species	Reference
Lsh	Resistance/susceptible (liver parasite load)	*L. mexicana*	[45, 48]
H2r, s, a, k	Lesion resorption	*L. major*, *L. tropica*, *L. mexicana*	[46–48]
H2b, d, q	Slow resolution of lesions	*L. major*, *L. tropica*, *L. mexicana*	[46, 48]
Scl1	Lesion healing	*L. major*	[16]
Scl2	Lesion development	*L. mexicana*	[16]
Lmr1, 2, 3….	Lesion healing	*L. major*	[50–52]

High throughput genetic screening revealed a complex picture. Indeed, some loci that control CL are common to several *Leishmania* spp., while others are specific. The *Lsh1* locus on chromosome 1 can control the outcome of cutaneous infections caused by *L. mexicana*, but not by *L. major* [44, 45]. The H2 locus also influences the infection outcome, but more weakly than the *Lsh1* locus. Depending on the HLA allele, mice have been classified as resistant (H2-r, -s, -a, -k) or susceptible (H2-b, -d, -q), with slowing resolving lesions following infection by *L. major*, *L. tropica* or *L. mexicana* [46–48].

Studies based on genome-wide analysis of resistance *versus* susceptibility phenotypes in the offspring of various intercrosses allowed the identification of different loci involved in mouse susceptibility or resistance to CL. For instance, the *scl*-1 and -2 (for susceptibility to cutaneous leishmaniasis) loci on chromosome 11 [49] are involved in the control of mouse susceptibility. The *scl*-*1* locus controls the healing *versus* non-healing responses to *L. major* and the scl-2 is responsible for the development of cutaneous lesions induced by *L. mexicana* [49]. On the other hand, others loci are involved in mouse resistance as the *Lmr*-1, -2 and -3 (for *Leishmania major* resistance) loci that contribute to control skin lesion healing (for review see: [50, 51]. Currently, more than 30 loci have been identified as involved in the complex control of *L. major*-induced CL. Indeed, a single Quantitative Trait Locus (QTL) does not account for the overall phenotype variance, but rather is responsible for the control of a specific infection or pathogenic aspect. For example, *Lmr*-5 regulates parasite load in spleen and *Lmr*-20 in lymph nodes, whereas *Lmr*-5 and *Lmr*-21 control the development of skin lesions [52]. Thus, contrary to VL where only two main loci (*Lsh1* and *H2*) are involved, CL outcome is regulated by the combination of several loci. The susceptibility/resistance of different mouse strains to CL is summarized in Table 4. The highly susceptible BALB/c mice and the resistant C57BL/6 J mice are widely used to study both the genetics and biology of the host response to CL.

**Table 4:** Susceptibility of mouse strains to cutaneous leishmaniasis

Phenotype	Mouse strain	Reference
Susceptible	C57BL/10 (B10), B10.D2/n, BALB/c, SWR/J, P/J, CcS-16	[46, 48, 52]
Resistant	A, AKR, B10.BR, B10.RIII, B10.D2, CBA, C3H, C57BL/6, DBA/2 J, STS, 129/Sv, CcS-5	[46–48, 52]

Due to the complex control of CL pathogenesis, we decided to focus mainly on *L. major* because it is the most studied strain. In experimental settings for *in vivo* studies, some parameters, described hereafter, need to be considered.

### Immune control of infection

Differently from VL induced by *L. donovani* complex species where a mixed Th1/Th2 response is observed during the infection course, the outcome of *L. major* induced-CL depends on the development of polarized Th1 or Th2 responses associated with resistance or susceptibility, respectively [53]. Indeed, in resistant mice (C57BL/6), a Th1-oriented immune response, associated with IFNγ, IL2 and IL12 production, is clearly observed. At the infection site, few parasites remain viable thanks to the presence of CD4^+^ CD25^+^ regulatory T cells that produce IL10 [54]. Conversely, susceptible mice (BALB/c) develop a Th2 immune response with IL4 production, leading to the development of uncontrolled lesions and disseminated visceral infection. Treg cells that produce IL4 and IL10 cytokines also play a role in disease promotion by expanding, or regulating the Th2 population [55]. In these mice, lesion severity is also associated with the production of IL17 that promotes neutrophil immigration and thus lesion progression [56].

In experimental mouse models of CL, several cell types are found in the lesions: neutrophils [57], macrophages [58], eosinophils [58], lymphocytes [59], mast cells [60] and NK cells [61]. These cells play a role during the different phases of the infection: (i) silent phase (no lesion formation or inflammation detection) with parasite invasion of skin resident macrophages and neutrophils; (ii) lesion development associated with migration and activation of cells of the innate immune system (mast cells, neutrophils, monocytes); (iii) lesion involution with migration of dendritic cells and T cells; and (iv) chronic phase characterized by lesion resolution and associated with parasite persistence, mainly in macrophages, and lifelong immunity [58].

Thus, immunity to *L. major* depends on multiple cell types that cooperate for the development of an effective and protective immune response. Understanding their respective role and how to modulate their function could lead to new therapeutic approaches for immunization and long-lasting protection. For example, as dendritic cell activation is required for protective immunity, vaccines using infected or antigen-loaded dendritic cells could lead to the development of a specific and efficient immune protective response.

The remaining part of this review will focus on the parameters that influence the mouse immune response to CL: parasite genotype, parasite dose, site of the intradermal inoculation (ear dermis, dorsal skin and hind footpad), and associated adjuvant (saliva).

### Parasite genotype

Few reports have assessed the natural virulence variability of different *L. major* strains in the same mouse model and the associated immune response. Li et al. compared the infection outcome of two clones derived from the same *L. major* strain. They found that the avirulence of the S2 clone, characterized by spontaneous lesion healing, was not correlated with its capacity to infect macrophages or the inoculated dose, but with parasite factors [62]. The study of the infection outcome in BALB/c mice inoculated with 19 *L. major* strains (12 from Tunisia, zymodeme 25; and 7 from the Middle East, zymodeme 26, 68, 70 or 103) revealed a large heterogeneity of disease severity (footpad lesion size) [63]. Interestingly, all Middle East strains presented high or intermediate virulence, whereas most Tunisian strains (10/12) showed lower virulence. This was correlated with their pathogenicity in humans. Higher virulence could be associated with a greater capacity to infect bone marrow-derived macrophages, faster growth in culture and the induction of a stronger Th2 response *in vivo*. Moreover, the *in vitro* study of two of these *L. major* clones (zymodeme 25) highlighted their different capacity of human dendritic cell invasion, a feature that could modulate the innate immune response [64].

The heterogeneity of lesion size in function of the parasite genotype was confirmed by another study in BALB/c mice [65]. In addition, these authors observed reproducible differences in lymph node parasite burden, depending on the *L. major* strain, at week 8 after inoculation. Specifically, the highest pathogenicity (based on the parasite load) was associated with induction of the Th2 immune response, whereas strains with intermediate or low pathogenicity elicited predominantly a Th1 immune response. Recently, the study of four Iranian strains in BALB/c mice highlighted their high diversity of lymph node parasite burden and cytokine expression and confirmed that the strain causing the lowest parasite burden induced mainly a Th1 response [66].

These results clearly demonstrate the importance of the parasite genotype in CL development, although one study suggested that high parasite dose could be the only important determinant of the Th1/Th2 response, independently of the parasite or the mouse genotypes [67].

### Parasite dose

Concerning the inoculated dose (Table 5), an initial study found that in susceptible BALB/c mice, no clinical sign was visible (such as increase in footpad size) following subcutaneous inoculation of low parasite doses (10^2^ to 10^3^ parasites). Conversely, inoculation of high doses (10^5^ to 10^7^ parasites) led to significant footpad enlargement [68]. This dose-dependent effect was confirmed in other studies. For example, lesions were apparent in all BALB/c mice injected with 10^4^ (MHOM/IR/-/173 strain) or 10^6^ (MHOM/IL/80/Friedlin strain) parasites [67]. Uzonna et al. confirmed that CL severity (asymptomatic with no lesion but IgG2a response > IgG1; apparent lesions with identical IgG1 and IgG2a levels; large lesions and even foot loss) in BALB/c mice depends on the injected dose [69]. Moreover, subclinically infected BALB/c mice are resistant to a secondary pathogenic infection (10^6^ parasites, footpad injection) and, thus, could be used in vaccination strategies [69]. However, not all mice infected with low parasite doses will develop a subclinical form of CL. Indeed, susceptible BALB/c mice inoculated with 10^2^ parasites showed significant pathology (antibody response and parasite detection in lymph nodes) and then progressed to a chronic phase where lesions stop increasing in size and eventually will resolve [69, 70]. In C57BL/6 mice, whatever the dose (10^2^ to 10^7^), lesions resolved and this was associated with the induction of a Th1 immune response (tested for the 10^2^ and 10^6^ doses) [70]. However, in another study, the classical clinical response (lesion healing and Th1 immune response) was observed only when C57BL/6 mice were inoculated with high parasite dose (10^6^). Conversely, in animals inoculated with low doses (10^3^), an unexpected, but transient Th2 response occurred first and was then reversed by the activation of IFNγ − producing CD8^+^ T cells [71].

**Table 5:** Influence of the parasite dose on cutaneous lesion size and the concomitant immune response

Parasite dose	Mouse strain	Parasite strain	Lesion size (footpad)	Th1 response	Th2 response	Reference
10^1^	BALB/c	MHOM/IL/80/Friedlin	+/-	+++	+/-	[69]
	BALB/c	MHOM/IR/-/173	+/-	ND	ND	[67]
10^2^	BALB/c	MHOM/IL/80/Friedlin	+/-	ND	ND	[68]
	BALB/c	MHOM/IL/80/Friedlin	-	++	+/-	[67]
	BALB/c	MHOM/IR/-/173	+	++	+/-	[67]
	BALB/c	MHOM/IL/80/Friedlin	+	+	-	[69]
	BALB/c	MHOM/IR/-/173	+/-	++	-	[70]
	C57BL/6	MHOM/IR/-/173	-			[70]
10^3^	BALB/c	MHOM/IL/80/Friedlin	+	-	+/-	[68]
	BALB/c	MHOM/IL/80/Friedlin	-	ND	ND	[67]
	BALB/c	MHOM/IL/80/Friedlin	++	+	+	[69]
	BALB/c	MHOM/IR/-/173	+	ND	ND	[70]
	C57BL/6	MHOM/IL/80/Friedlin	+	+	++	[71]
	C57BL/6	MHOM/IR/-/173	-	ND	ND	[70]
10^4^	BALB/c	MHOM/IL/80/Friedlin	+/++	ND	ND	[68]
	BALB/c	MHOM/IL/80/Friedlin	+	ND	ND	[67]
	BALB/c	MHOM/IR/-/173	++	ND	ND	[67]
	BALB/c	MHOM/IL/80/Friedlin	++	+/-	+	[69]
	BALB/c	MHOM/IR/-/173	+	ND	ND	[70]
	C57BL/6	MHOM/IR/-/173	+/-	ND	ND	[70]
10^5^	BA[71]LB/c	MHOM/IL/80/Friedlin	++	ND	ND	[68]
	BALB/c	MHOM/IL/80/Friedlin	+++	+/-	++	[69]
	BALb/c	MHOM/IR/-/173	+	ND	ND	[70]
	C57BL/6	MHOM/IR/-/173	+/-	ND	ND	[70]
10^6^	BALB/c	MHOM/IL/80/Friedlin	+++	-	++	[68]
	BALB/c	MHOM/IL/80/Friedlin	++	ND	ND	[67]
	BALB/c	MHOM/IL/(0/Friedlin	+++	+/-	+++	[69]
	BALB/c	MHOM/IR/-/173	++	+	+	[70]
	C57BL/6	MHOM/IL/80/Friedlin	+	++	-	[71]
	C57BL/6	MHOM/IR/-/173	+	++	-	[70]
10^7^	BALB/c	MHOM/IL/80/Friedlin	++++	+/-	++	[68]
	BALB/c	MHOM/IL/80/Friedlin	+++	+/-	++	[67]
	BALB/c	MHOM/IR/-/173	++	ND	ND	[70]
	C57BL/6	MHOM/IR/-/173	+	ND	ND	[70]

In summary, the inoculated dose can influence the immune response and thus CL severity, which is also dependent on the mouse genetic background. Specifically, in susceptible mice (i.e. BALB/c strain), high doses (10^5^ to 10^7^ parasites) lead to persistent infection associated with a Th2 immune response. Low doses (10^1^ or 10^2^ parasites) induce a Th1 response and thus could be used in vaccination studies. However, a “serodeconversion” may occur several (9 to 18) months after infection. With intermediate doses (10^3^-10^4^), a mixed Th1-Th2 immune response is observed.

In resistant mice (i.e., C57BL/6 strain), effective lesion development is observed only with high doses (10^6^-10^7^). Inoculation of low doses (100 metacyclic promastigotes) at a dermal site (for instance, ear dermis) is recommended to mimic the natural transmission and induces two distinct disease phases [58, 72]. First, a clinically silent phase occurs during the first 4–6 weeks and is characterized by the absence of lesions and the increase of the parasite load. During the second phase, lesions develop (footpad swelling) associated with immune cell infiltration at the site of infection. Concomitantly, Th1 cells expand in the draining lymph nodes, ultimately leading to parasite burden reduction and lesion healing.

### Inoculation site

In experimental studies on CL, only the intradermal route of inoculation is used, but at different sites: hind footpad, ear pinna and tail base (Table 6). Depending on the inoculation site, the clinical signs (lesion size and immune response) clearly differ. Moreover, the cytokine production profile does not always explain the disease severity [73, 74].

**Table 6:** Influence of the inoculation site on cutaneous lesion size and immune response

Inoculation site	Mouse strain	Lesion size	Th1 response	Th2 response	Reference
Hind footpad	BALB/c	+++	+	+++	[73]
	SWR	+	+++	+	
	C57BL/6J	+/-	ND	ND	
Ear pinna	BALB/c	+++	-	++	[74]
	C57BL/6J	+	++	-	
	DBA/2	+/-	-	+/-	
	C3H/HeN	-	+/-	+	
	CBA/H	-	-	+/-	
Tail base	BALB/c	++	-	++	[74]
	C57BL/6J	+/-	+	+	
	DBA/2	++	-	-	
	C3H/HeN	+	++	-	
	CBA/H	+/-	-	-	
	BALB/c	++	+	+++	[73]
	C57BL/6J	-	ND	ND	
	SWR	++	+	+/-	

In BALB/c mice, severe, non-healing lesions were observed whatever the inoculation site [73, 74], and they were associated with a classical Th2 immune response [73, 74].

In SWR mice, inoculation at the base of the tail led to the development of large non-healing lesions, whereas self-healing lesions were observed following inoculation in the hind footpad [73]. Unexpectedly, inoculation at the tail base tail induced a Th1 immune response, which is normally associated with self-healing lesions.

This dichotomy was also observed in resistant mice, such as the C57BL/6J strain. After inoculation in the ear pinna, the classical Th1 response was associated with the development of small, self-healing lesions. Conversely, parasite inoculation at the tail base, induced a Th2 response that was unexpectedly associated with lesion healing [74].

C3H/HeN and DBA/2 mice were resistant to CL caused by ear pinna inoculation of *Leishmania* parasites, but presented intermediate disease (C3H) or were fully susceptible (DBA/2) when inoculated at the tail base [74]. Again, no clear correlation was found between the type of immune response and the cutaneous lesion severity. For instance, C3H/HeN mice were fully resistant to *L. major* infection through the ear pinna, but no Th1 response could be detected.

CBA/H mice were resistant to *L. major* infection through any inoculation site, with an immune response similar to controls [74].

Thus, besides the parasite dose and mouse genetic background, the infection site also affects CL severity.

### Influence of phlebotome saliva

Several studies assessed the role of the vector saliva in CL development.

When *L. major* parasites were inoculated with saliva from *Lutzomyia longipalpis*, the size of cutaneous lesions in CBA and BALB/c mice was five to ten times bigger and contained at least 5000 times more parasites than in controls (no saliva) [75]. Moreover, when inoculated at low doses, parasites survived only when co-injected with saliva [75]. This disease exacerbation effect was observed in other mouse strains (susceptible, intermediate susceptible or resistant) and was more pronounced in resistant CBA and C57BL/6 mice [76]. Salivary gland extracts from *L. longipalpis* exacerbated CL following infection by *L. major* [78] or by *L. braziliensis* [57, 78]. It has been demonstrated that maxadilan, a salivary vasodilator, is responsible for the disease-exacerbation effect of saliva from *L. longipalpis* [79].

*Phlebotomus papatasi* saliva also can increase lesion size, but with a less pronounced effect than saliva from *L. longipalpis* [76]. Moreover, inoculation of parasites with *P. papatasi* saliva in resistant CBA mice led not only to bigger lesion size, but also to higher parasite burden in lesions that was associated with a modulation of the immune response (decrease of Th1 factors and increase of Th2-associated IL4 production) [80]. When mimicking natural infection by injection of low dose of parasites in the ear dermis, co-inoculation of *P. papatasi* saliva promoted lesion development (earlier and higher parasitemia) in BALB/c and also in C57BL/6 mice [81]. This was associated with Th2 immune response induction and IL4 production.

### Additional experimental parameters

To our knowledge, only one study in BALB/c mice described the influence of *L. major* infectious stage on (i) lesion development and ulceration and (ii) on the type of immune response [82]. Specifically, after inoculation of high doses (10^6^) of metacyclic promastigotes (infective stage) in the ear dermis (pinna), lesions were detectable 1 week after infection and became ulcerate after 4 weeks. Following inoculation of high doses (10^6^) of log-phase parasites (division stage), detectable lesions and ulcerations were observed after three and 10 weeks, respectively. Moreover, inoculation of log-phase parasites led to better activation of lymph node CD4^+^ T cells (IFNγ production) than inoculation of metacyclic promastigotes, at least during the early stages of infection (16 h and 3 days post-inoculation).

In addition, the presence of apoptotic promastigotes in the infectious inoculum is important for the parasite intracellular survival and, thus, for disease development *in vivo* [83]. Although this parameter must be further investigated, it has to be taken into account when designing *in vivo* experiments.

In summary, when studying CL, the route of infection, the infectious parasite stage, the inoculated dose or the adjuvant, the mouse resistance or susceptibility to infection are all essential parameters to be taken into account because they can substantially influence the issue of *in vivo* experiments.

## Conclusions

In conclusions, this review underlines that many parameters have to be taken into account for the *in vivo* study of *L. donovani* complex or *L. major* infection in mouse models of VL and CL.

From the host genetic point of view, it seems more complex controlling CL than VL. Indeed, several loci are involved in CL control and each locus regulates specific features of the disease (i.e., IgG secretion, parasite load, lesion size). Although the use of an animal model limits the influence of the environment, the choice of the mouse genetic background is crucial. For instance, a sensitive mouse strain is more suitable for comparing the infection outcome of various *Leishmania* strains and for rapid assessment of the parasite virulence and/or pathogenicity.

Concerning the immune response (resistance/susceptibility phenotype), in VL, mice are clearly classified according to their capacity to sustain parasite persistence in organs. In CL, parasites could persist at the infection site in resistant mice, thus giving a lifelong immunity to reinfection.

For both CL and VL, the parasite genotype clearly affects the infection outcome; however, it is difficult to correlate the results obtained in animal models with clinical observations in human patients.

The parasite dose and the route of inoculation need also to be carefully considered.

Inoculation through the intradermal route must be performed with high dose of parasite inoculum to give effective viscera infection during VL, whereas low doses can be used for CL with parasites dissemination to visceral organs in susceptible mice. In the case of *L. infantum* infection, different immune responses are observed in function of the inoculation route and this can strongly influence the outcome. Thus, to study the chronic phase of the infection, an inoculation route that promotes the parasite long-term establishment (intravenous route) must be preferred. Conversely, when studying the acute phase, the inoculated dose (high doses leading to more intense immune response) is the crucial point. For *L. major*, the key parameters seem to be the dose and its association with sandfly saliva. Low doses are recommended for immunization studies, but not for strain virulence assessment. Indeed, at low doses, lesion development is controlled in susceptible mice, except when saliva is simultaneously injected. Effective lesion development requires inoculation of high doses, leading to the development of ulcers the severity of which is directly correlated with the strain virulence.

## Abbreviations

CL: Cutaneous leishmaniasis
IFNg: Interferon gamma
IL: Interleukin
Lmr: *Leishmania major* resistance
NK: Natural killer
NO: Nitric oxyde
QTL: Quantitative Trait Locus
scl: Susceptibility to cutaneous leishmaniasis
TGF: Transforming growth factor
TNF: Tumor necrosis factor
Treg: Regulatory T cells
VL: Visceral Leishmaniasis
W.H.O: World Health Organization
